# Regulation of Ptch1 by miR-342-5p and FoxO3 Induced Autophagy Involved in Renal Fibrosis

**DOI:** 10.3389/fbioe.2020.583318

**Published:** 2020-10-29

**Authors:** Simin Tang, Yi Wang, Guiling Xie, Wenjun Li, Yanna Chen, Jinshu Liang, Pei Liu, Fuhu Song, Jun Zhou

**Affiliations:** Department of Anesthesiology, Third Affiliated Hospital, Southern Medical University, Guangzhou, China

**Keywords:** renal fibrosis, Ptch1, miR-342-5p, FOXO3, bioinformatics, autophagy

## Abstract

The pathogenesis of renal fibrosis (RF) is not well understood. Here, we performed an integrative database analysis of miRNAs and mRNAs to discover the major regulatory pathway in RF. Putative miRNAs and mRNAs involved in RF in unilateral ureteral obstruction (UUO) model mice were extracted and analyzed using the Gene Expression Omnibus (GEO) database. The bioinformatics analysis suggested that Ptch1 expression is regulated by miR-342-5p and FoxO3. Then real-time PCR, Western blot, Fluorescence *in situ* hybridization were done to confirm the hypothesis. Sixty-three differentially expressed miRNAs (DE-miRNAs) in GSE118340, 141 DE-miRNAs in GSE42716, and 183 DE-mRNAs in GSE69101 were identified. Various bioinformatic analyses revealed miR-342-5p as a strong candidate regulator in RF. We also predicted that miR-342-5p targets Ptch1 and that FoxO3 is the transcription factor of Ptch1. We also observed that TGF-β1 upregulated the expression of miR-342-5p and inhibited the expression of FoxO3 and Ptch1 in TCMK-1 cells. Furthermore, downregulation of miR-342-5p reversed the inhibitory effect of TGF-β1 on the expression of Ptch1 in TCMK-1 cells, while downregulation of FoxO3 promoted the inhibitory effect of TGF-β1 on the expression of Ptch1. Additionally, downregulation of Ptch1 increased TGF-β1-induced autophagy, as evidenced by an increase in the number of GFP-LC3 puncta and the increased protein expression of SOSTM1/p62 and LC3II/LC3I. Our findings showed that Ptch1 expression is negatively regulated by miR-342-5p and positively regulated by FoxO3, and downregulation of Ptch1 induced autophagy in TGF-β1-stimulated TCMK-1 cells. These findings will further our understanding of the molecular mechanisms of RF and provide useful novel therapeutic targets for RF.

## Introduction

As the terminal pathological alteration of almost all progressive kidney diseases, renal fibrosis (RF) is characterized by hyperproliferation, activation of fibroblasts, and increased deposition of extracellular matrix (ECM), thereby distorting normal kidney tissue architecture and causing loss of kidney function (Boor et al., 2010). There is an urgent unmet clinical need for effective therapies and understanding of the molecular pathogenesis of RF. MicroRNAs (miRNAs) are endogenous small non-coding 21–25 nucleotide single-stranded RNAs. miRNAs regulate gene expression by promoting the degradation of mRNAs or inhibiting the translation of mRNAs. miRNAs are involved in the development of various diseases, and several miRNAs are closely associated with RF, including miR-184, miR-34a, and miR-199a-3p (Yang et al., 2017; Chen, 2019; Liu et al., 2019). Our review showed the recent progress, biological functions, and clinical applications of miRNAs in the progression of RF (Fan et al., 2020). However, the role of most miRNAs in RF is still unclear. Autophagy is a highly evolutionarily conserved lysosomal degradation pathway that plays a vital role in maintaining cellular homeostasis (Klionsky, 2007). Autophagy dysfunction is implicated in the pathogenesis of acute kidney injury and RF diseases (Hartleben et al., 2010; Jiang et al., 2012; Nam et al., 2019). In the past few years, miRNA-mediated autophagy has been reported to be involved in RF (Li X.Y. et al., 2017; Nam et al., 2019; Yang et al., 2020). However, the specific mechanism by which miRNA-mediated autophagy regulates RF has not been fully elucidated.

Obstructive ureteral mechanical damage is the primary clinical etiology of RF. Exploring the genetic changes in the obstructive kidneys helps to understand the molecular and cellular mechanisms involved in the pathogenesis of RF. Unilateral ureteral obstruction (UUO) is a classic animal model that can simulate obstructive nephropathy (Chevalier et al., 2009). Recently, with the rapid development of microarray and high-throughput sequencing technologies, the mechanisms of RF at the genomic level have been increasingly explored (Lee et al., 2014; Zhou et al., 2018; Higashi et al., 2019). We analyzed two comprehensive and integrated miRNA datasets (GSE118340 and GSE42716) and one mRNA dataset (GSE69101) from the Gene Expression Omnibus (GEO) database. Two representative DE-miRNAs (miR-342-5p and miR-466a-3p) were shared and extracted for further analysis. Previous studies have reported that miR-342-5p was strictly related to the deposition of ECM, inflammatory cytokine release, and the TGF-β signaling pathway (Wei et al., 2013; Yan et al., 2016; Qu et al., 2019), which are involved in the mechanisms of RF. However, the mechanisms by which miRNAs mediate the specific molecular changes observed in RF have not been studied. There are no studies on the role of miR-342-5p in the development of RF. Thus, we further focused on the mechanism by which miR-342-5p is involved in RF progression.

Combined with in-depth bioinformatics analysis, we predicted that miR-342-5p directly targets Ptch1, which is an endogenous inhibitor of pro-fibrotic signaling pathways, and is regulated by the transcription factor (TF) FoxO3. Herein, we verified the relationship between miR-342-5p, FoxO3, and Ptch1 *in vitro*. Our results indicated that miR-342-5p expression was upregulated in TCMK-1 cells stimulated with TGF-β1, while FoxO3 and Ptch1 were downregulated. Rescue experiments showed that the downregulation of miR-342-5p reversed the inhibitory effect of TGF-β1 on Ptch1 expression in TCMK-1 cells, while the inhibition of FoxO3 promoted the inhibitory effect of TGF-β1 on Ptch1 expression. In addition, autophagy was increased in response to TGF-β1, and knockdown of Ptch1 in TCMK-1 cells increased autophagy markers. Collectively, these data suggest that miR-342-5p and FoxO3 regulate Ptch1, which mediates autophagy involved in the RF process. These findings further the understanding of the molecular mechanisms of RF and provide therapeutic targets for RF diseases.

## Materials and Methods

### Microarray Dataset

Two miRNA datasets, GSE118340 and GSE42716, were downloaded from the GEO database^1^. GSE11840 was based on the GPL19057 (Illumina NextSeq 500), and GSE42716 was based on the GPL10384 [Agilent-021828 Unrestricted Mouse miRNA Microarray (V2)]. In total, four kidney samples of the UUO model (GSM3325596, GSM3325597, GSM3325598, and GSM3325599) and three kidney samples of the sham control (GSM3325589, GSM3325590, and GSM3325591) from GSE118340 were selected for analysis. Four kidney samples of the UUO model (GSM1048441, GSM1048442, GSM1048443, and GSM1048444) and four kidney samples of the sham control (GSM1048437, GSM1048438, GSM1048439, and GSM1048440) from GSE42716 were also selected. The mRNA dataset GSE96101 was also downloaded from the GEO dataset, which is based on the GPL4134 (Agilent-014868 Whole Mouse Genome Microarray 4 × 44K G4122F). Six kidney samples of the UUO model (GSM2533412, GSM2533413, GSM2533414, GSM2533415, GSM2533416, and GSM2533417) and five kidney samples of the sham control (GSM2533473, GSM2533474, GSM2533475, GSM2533476, and GSM2533477) were selected for analysis. The above kidney samples were all harvested from mice 7 days after UUO surgery and then were subjected to microarray analysis of miRNAs and mRNAs. Contralateral kidneys without ureteral obstruction were used as controls. Subsequently, data quality from these datasets was assessed using R software.

### Identification of Differentially Expressed miRNAs (DE-miRNAs) and mRNAs (DE-mRNAs)

The R software “DESeq2” package was used to identify DE-miRNAs in kidney tissues between UUO and sham control (Love et al., 2014). DE mRNAs were identified using the online tool GEO2R^2^, which allows users to compare two or more datasets in the GEO series to determine DEGs under experimental conditions (Davis and Meltzer, 2007). Genes with more than one probe set were removed. In the present study, DE-miRNAs and DE-mRNAs were combined using the cut-off point of adj.*P*-value < 0.05, and | logFC| > 1.0. Then, the R heatmap and ggplot2 package were applied to paint the heatmap and Volcano. adj.*P* < 0.05 means statistical significance.

### GO Annotation and KEGG Pathway Enrichment Analysis

The selected DE-miRNAs were then uploaded to miRpath v3.0^3^, an online website that integrates miRNA target gene databases, including TarBase, TargetScan, and microT-CDS (Vlachos et al., 2015). It only needs to input the miRNA Id of interest and obtain the results of Gene Ontology (GO) and Kyoto Encyclopedia of Gene and Genome (KEGG) enrichment analysis. The selected DE-mRNAs were then uploaded to DAVID^4^, a database that conducts functional annotation bioinformatics microarray analysis (Huang da et al., 2009). The top 10 GO annotations and KEGG pathways were visualized using GraphPad Prism.

### miRWalk and Targetscan

miRWalk^5^ can predict the mRNAs targeted by specific miRNAs using a machine learning algorithm. Thus, this database was used to predict the targeted mRNAs of miR-342-5p. Targetscan 7.2^6^, an online database that is currently used to predict the location of miRNA binding sites with an accuracy of up to 90%, was applied to revalidate the binding sites of miRNA and mRNA.

### Comparative Toxicogenomics Database (CTD)

Comparative Toxicogenomics Database^7^ is a powerful, publicly available database designed to advance understanding of how environmental exposures affect human health and to clarify the relationship among genes, drugs, and diseases. We used this database to evaluate the inference score of selected mRNAs in chronic kidney diseases (CKDs).

### The iRegulon

The iRegulon, a Cytoscape App that consists of a TF and its direct transcriptional targets, which contain common TF binding sites in their cis-regulatory control elements. It predicts TFs by calculating motif enrichment analysis. The motif enrichment analysis applies multiple position weight matrices (PWM), and finally sorts each motif. The preferred motif was used to predict the final TFs (Janky et al., 2014).

### Cell Culture and Treatment

Mouse kidney epithelial cell line TCMK-1 was cultured in RPMI-1640 (Hyclone, Cat. No. SH30809.01B) supplemented with 10% fetal bovine serum (Hyclone, Cat. No. SH30087.01) and 1% penicillin-streptomycin (Hyclone, Cat. No. SH30010) at 37°C under saturated humidity and 5% CO_2_ conditions. The cells were starved for 12 h; on reaching 70% confluence, TGF-β1 (eBioscience, 14-8342-82) was added to the medium at a final concentration of 10 ng/ml for 0, 1, 2, 4, 8, 12, and 24 h, to choose suitable time points for the follow-up experiment.

### Cell Transfection

TCMK-1 cells were passaged 1 day before transfection to obtain 30–50% confluence. Transfection was performed using Lipofectamine^TM^ RNAiMAX (Invitrogen, Cat. No. 13778075), and the miRNA/siRNA working concentration was 50 nM using Opti-MEM (Invitrogen, Cat. No. 31985070). In LC3 plasmid cell transfection, TCMK-1 cells were passaged 1 day before transfection to obtain 70–80% confluence. Transfection was performed using Lipofectamine 2000 (Invitrogen, Cat. No. 11668019), Premo^TM^ Autophagy Sensor LC3B-RFP (BacMam 2.0 Invitrogen, P36236), and Opti-MEM (Invitrogen, Cat. No. 31985070). Approximately four h after transfection, the transfection mixture was replaced with DMEM-10% FBS. The punctate distribution of GFP-LC3 was observed using fluorescence microscopy. The remaining cells were then harvested for RNA and protein preparation.

### Quantitative Real-Time PCR

Total RNA of TCMK-1 cells was extracted with TRIzol^®^ Reagent (Invitrogen, 15596026) according to the manufacturer’s protocol. DNA was removed by treating with DNase (Fermentas, EN0521) and the purity of the RNA was assessed by determining the absorbance ratio at OD260/OD280. Total RNA was reverse transcribed into cDNA using TaKaRa PrimeScript II 1st Strand cDNA Synthesis Kit (TaKaRa, D6210A), Oligo dT Primer, dNTP Mixture, total RNA, and RNase Free dH_2_O. Real-time PCR was performed using the TaKaRa SYBR^®^ Premix Ex TaqTM II kit (Perfect Real Time), and ABI PRISM^®^ 7500 Sequence Detection System Real-Time PCR System. Primer sequences for the TCMK-1 cell samples were as follows:

FoxO3-F: 5′ - AACCGGCTCCTTCAACAGTA
FoxO3-R: 5′ GAAGCAAGCAGGTCTTGGA
Ptch1-F: 5′ - TCTGCTTCGGTGACTGTTG
Ptch1-R: 5′ - CCACGTCCTGTAGCTCTATG
β-actin-F: 5′ GCTTCTAGGCGGACTGTTAC
β-actin-R: 5′ CCATGCCAATGTTGTCTCTT
miR-342-5p-F: 5′ ACACTCCAGCTGGGAGGGGTGCTA TCTGTGAT
miR-342-5p-R: 5′ CTCAACTGGT GTCGTGGA
miR-342-5p-RT:5′ CTCAACTGGTGTCGTGGAGTCGG CAATTCAGTTGAGCTCAATCA
U6-F:5′ - CTCGCTTCGGCAGCACA
U6-R:5′ AACGCTTCACGAATTTGCGT
U6-RT: 5′ AACGCTTCAC GAATTTGCGT

Reactions to determine the levels of Ptch1 and FoxO3 were carried out in a total volume of 20 μL, including 10 μL SYBR Premix Ex Taq TM (2×), 2 μL cDNA, 6.4 dH_2_O, and 0.8 μL of each specific primer (10 μM), while the reaction for miR-342-5p was carried out in a total volume of 8 μL, including 2 μL 10 mM dNTP (Promega), 0.5 μL RNase inhibitor (Promega), 0.5 μL miR-342-5 primer, 0.5 μL U6 primer, 4 μL 5× buffer, and 0.5 μL M-MLV (Promega). The cycling conditions were set at 94°C for 3–4 min; 30 cycles at 94°C for 30 s, 60°C for 30 s, and 72°C for 30 s; the last cycle (1×) was set at 72°C for 10 min and 16°C for ∞. β-actin was utilized for standardization of Ptch1 and FoxO3, and U6 was used as the endogenous control for miR-342-5p. The levels of mRNAs and miRNAs were calculated using the 2^–Δ^ ^Δ^ Ct method with the ABI PRISM^®^ 7500 Sequence Detection System SDSShell Software 1.6.

### Western Blot

Protein samples were extracted with RIPA buffer. The extracted total protein was separated by SDS-PAGE and transferred to PVDF membranes. Following incubation in blocking buffer for 1 h at room temperature, the membrane was incubated at 4°C overnight with Ptch1 antibody (Abcam, ab109096), Foxo3 antibody (Thermo Fisher Scientific, FA1-14171), SQSTM1/p62 antibody (Abcam, ab56416), and LC3B (D11) XP antibody (Cell Signaling Technology, 3868S). The results were visualized using Beyo ECL Plus substrate and exposed to X-ray film. β-actin was used for normalization.

### Fluorescence *in situ* Hybridization

Fluorescence *in situ* hybridization was performed to detect the location of miR-342-5p, FoxO3, and Ptch1. We used a miR-342-5p probe labeled with FAM (green) and FoxO3 and Ptch1 probes labeled with cy3 (red). The cells were then visualized using a scanning laser confocal microscope (Leica, TCS SP2 AOBS).

### Statistical Analyses

All data are shown as the mean ± SD and visualized using GraphPad Prism 8. Statistical analysis was conducted using SPSS 24.0. *P* < 0.05 was considered significant. All experiments were performed at least three times.

## Results

### Identification of Candidate DE-miRNAs

**FIGURE 1:**
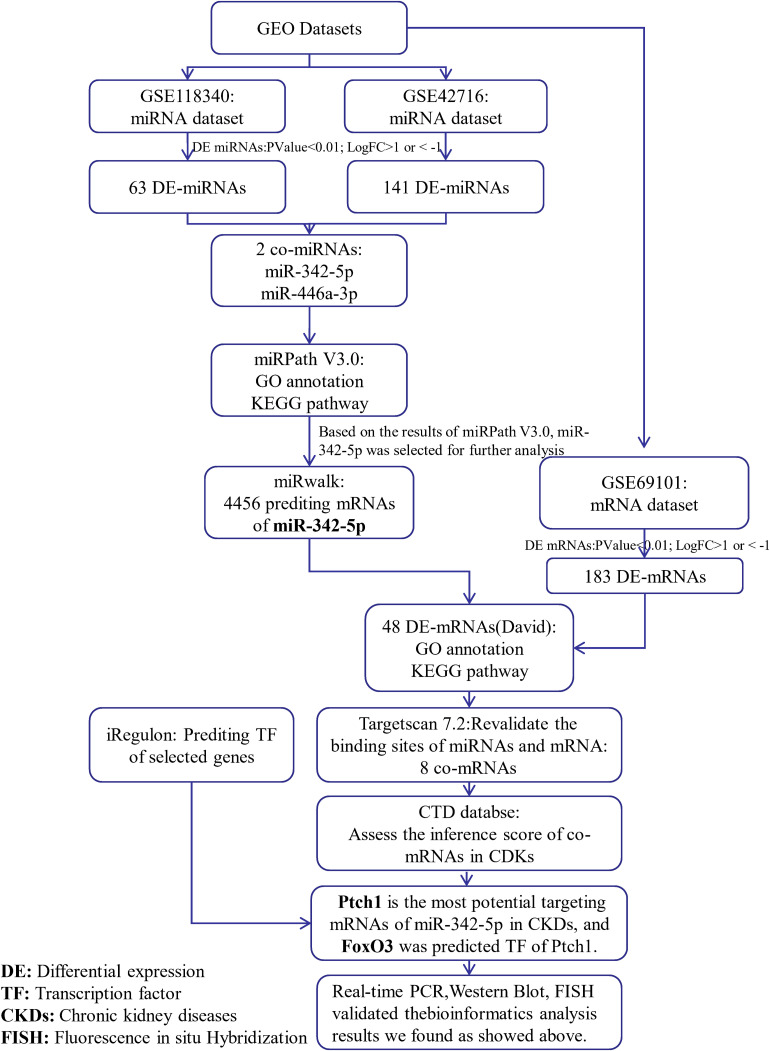
Simple flow chart of bioinformatics analysis and experimental design.

**FIGURE 2:**
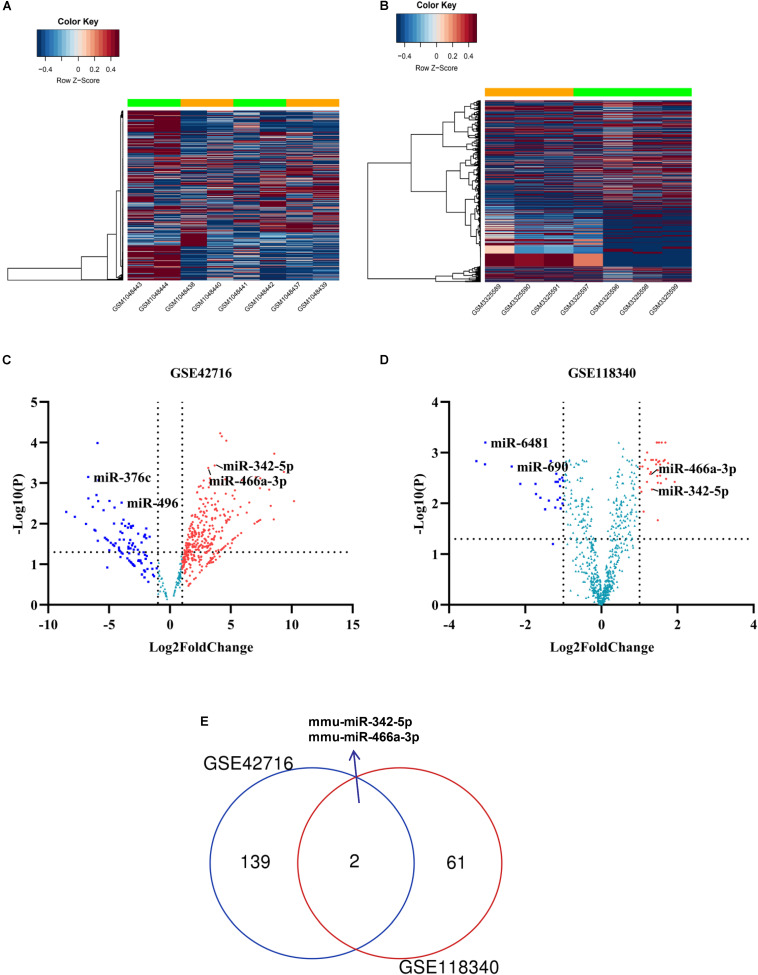
Candidate DE-miRNAs were obtained from GSE118340 and GSE42718. **(A,B)** Heatmap showing the expression levels of each gene in different groups from GSE118340 and GSE42718. **(C,D)** Volcano plot showing the DE-miRNAs in GSE118340 and GSE42718. **(E)** Venn diagram showing miR-342-5p and miR-466a-3p are common DE-miRNAs in both GSE118340 and GSE42718.

To identify potential miRNA-mRNA regulatory networks in RF, the datasets GSE118340 and GSE42716 were selected to screen DE-miRNAs between UUO samples and normal samples. And the simple flow chart of bioinformatics analysis and experimental design were showed (Figure 1). Briefly, the distribution of gene expression data in each group was revealed in the heatmap and unsupervised clustering analysis (Figures 2A,B). Sixty three DE-miRNAs and 141 DE-miRNAs (adj.*P* < 0.05, | logFC| > 1) were detected from GSE118340 and GSE42716 using DESeq2 package analysis. A volcano plot was used to visualize the DE-miRNAs from the two datasets (Figures 2C,D). The Venn diagram showed that only two DE-miRNAs (miR-342-5p and miR-466a-3p) were shared in both the GSE118340 and GSE42716 datasets and were extracted for further analysis (Figure 2E).

### GO Annotation and KEGG Pathway Enrichment Analysis

**FIGURE 3:**
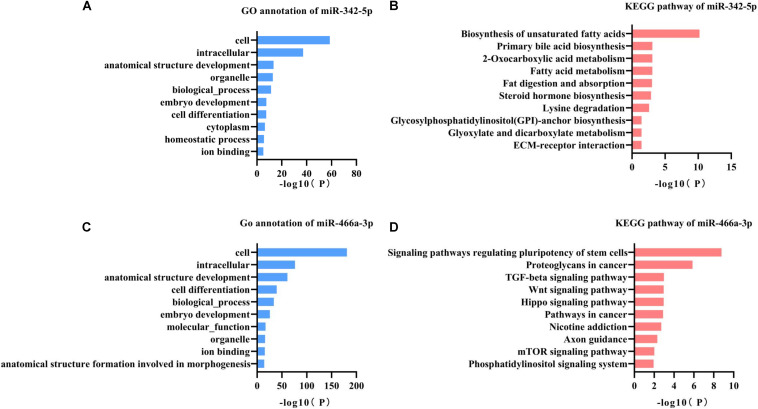
GO annotation and KEGG pathway analysis of miR-342-5p and miR-466a-3p. **(A,B)** GO annotation and KEGG pathway analysis of miR-342-5p obtained from miRPath V3.0. **(C,D)** GO annotation and KEGG pathway analysis of miR-466a-3p obtained from miRPath V3.0.

miR-342-5p and miR-466a-3p relevant GO annotation and KEGG pathways were identified using the mirPath v3.0 web server, and the results showed that 39 GO and 13 KEGG pathways were significantly (*P* < 0.05) regulated by miR-342-5p, while 36 GO and 13 KEGG pathways (Supplementary Tables S1, S2) were significantly regulated by miR-466a-3p. The top 10 GO annotations and KEGG pathways of miR-342-5p and miR-466a-3p are shown in Figures 3A–D. All the above identifications were based on merging and meta-analysis algorithms according to experimentally validated miRNA target interactions. The pathways regulated by the miR-342-5p closely related to the pathophysiology of RF should be involved in ECM-receptor interaction; whereas, miR-466a-3p is closely associated with the TGF-β signaling pathway.

### Identification of DE-mRNAs

**FIGURE 4:**
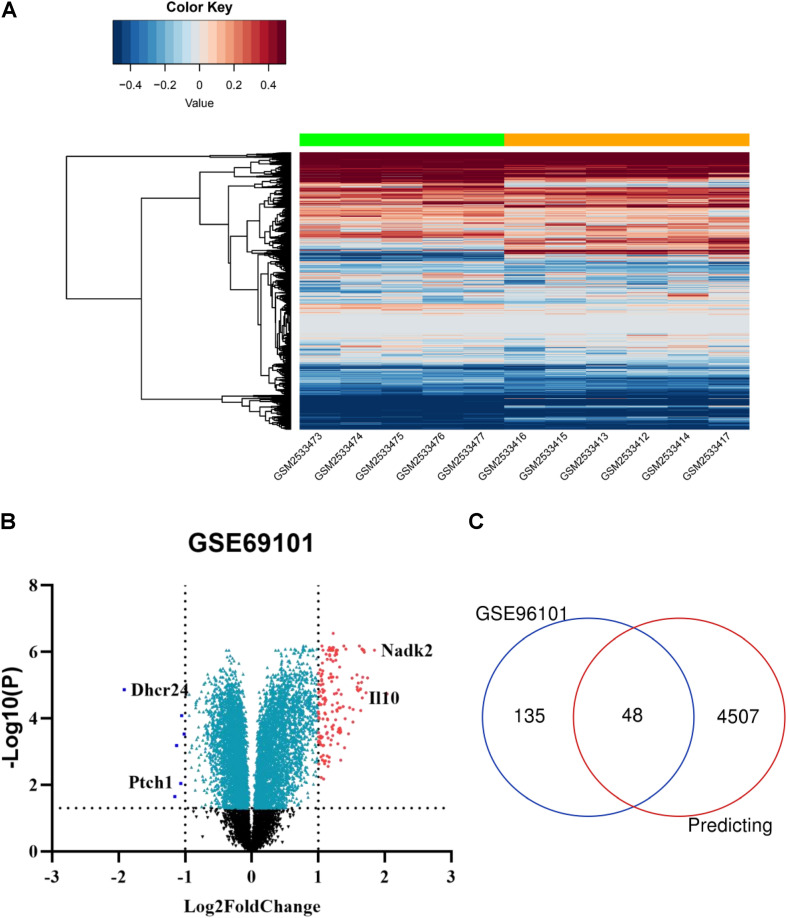
Candidate DE-mRNAs obtained from GSE96101. **(A)** Heatmap showing the expression levels of each gene in different groups from GSE96101. **(B)** Volcano plot showing the DE-mRNAs in GSE96101. **(C)** Venn diagram showing 48 common DE mRNAs obtained from GSE96101 and predicted mRNAs from miRWalk.

Using the GEO2R online tool, 183 DEGs (adj.*P* < 0.01, | logFC| > 1) were detected in the GSE96101, including 177 upregulated and 6 downregulated DEGs. In addition, a volcano plot and a heatmap of all DE-mRNAs were generated using the R ggplot2 package (Figures 4A,B). Then, mRNAs obtained from miRwalk and DE-mRNAs extracted from GSE96101 were subjected to Venn analysis. Venn diagram showing 48 mRNAs were overlapped and are listed below in detail (Figure 4C). GO annotation and KEGG pathways were obtained from DAVID and are summarized in Supplementary Table S3. We found that Ptch1 was mainly involved in intracellular protein transport, protein localization, and protein processing.

### Prediction of miR-342-5p and FoxO3 Correlation With Ptch1

**FIGURE 5:**
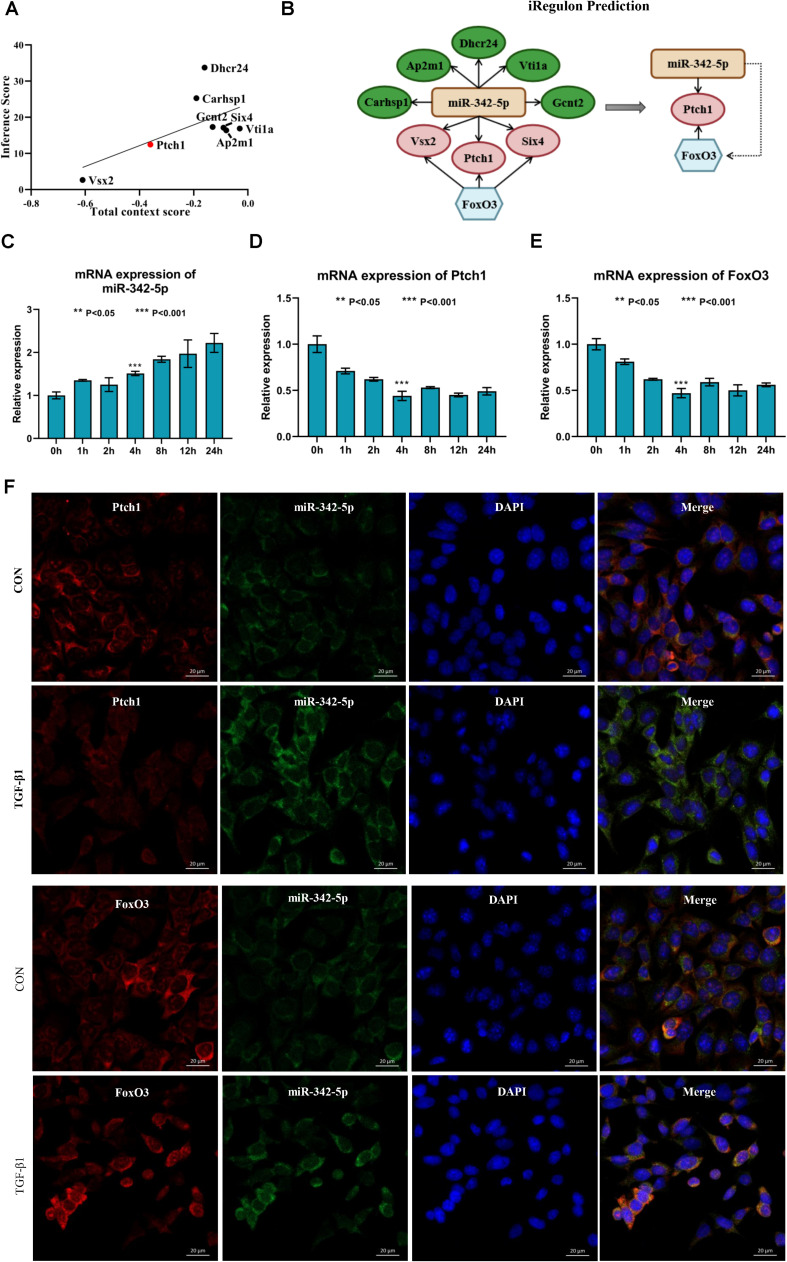
Ptch1 is the most promising target mRNA of miR-342-5p. **(A)** Regression analysis showing Ptch1 as the most promising target mRNA of miR-342-5p. **(B)** The iRegulon prediction showing FoxO3 as potential transcription factor of Ptch1. **(C–E)** Real-time PCR showing that TGF-β1 increases miR-342-5p and decreases FoxO3 and Ptch1 in TCMK-1 cells. **(F)** FISH experiments showing that miR-342-5p is mainly distributed in the cytoplasm of TCMK-1 cells, as is FoxO3 and Ptch1. The merged images show that miR-342-5p is partially colocalized with FoxO3 and Ptch1.

Next, we used Targetscan 7.2 to revalidate the binding sites of miRNAs and mRNAs, further narrowing the scope of the interaction of miRNA-mRNA. We found that only 8 DE-mRNAs were interacted with to miR-342-5p. Among them, Vsx2 and Ptch1 had the higher context score (Table 1). Then, the CTD database was used to assess the inference score of these 8 DE-mRNAs in CKDs. The results showed that Dhcr24 and Carhsp1 had higher inference scores (Table 2). Combining the results obtained from these two databases, we used regression to calculate and select the most suitable target genes of miR-342-5p. The results showed that Ptch1 is the most promising target mRNA for miR-342-5p (Figure 5A). Furthermore, we used the iRegulon to predict the TFs of the network. The results showed that FoxO3 is a potential TF of Ptch1 (Figure 5B).

**TABLE 1:** Targetscan7.2 predicts the mRNA targets of miR-342-5p.

Target gene	Representative transcript	3P-seq tags + 5	Total sites	8mer sites	7mer-m8 sites	7mer-A1 sites	6mer sites	Cumulative weighted context score	Total context score
Vsx2	ENSMUST00000021665.6	5	2	2	0	0	1	−0.61	−0.61
Ptch1	ENSMUST00000021921.5	4095	1	1	0	0	1	−0.36	−0.36
Gcnt2	ENSMUST00000110191.3	49	1	0	0	1	1	−0.13	−0.13
Six4	ENSMUST00000043208.7	266	1	0	0	1	1	−0.09	−0.09
Dhcr24	ENSMUST00000047973.3	9729	2	0	0	2	1	−0.08	−0.16
Vti1a	ENSMUST00000095950.2	693	1	0	0	1	0	−0.02	−0.03
Ap2m1	ENSMUST00000007216.8	2635	1	0	0	1	0	0	−0.08
Carhsp1	ENSMUST00000008537.8	1872	1	0	1	0	0	0	−0.19

**TABLE 2:** CTD database assesses the inference score of targeting mRNAs in chronic renal disease.

Gene symbol	Disease name	Direct evidence	Inference score
Dhcr24	Kidney failure, chronic	0	33.74
Carhsp1	Kidney failure, chronic	0	25.27
Gcnt2	Kidney failure, chronic	0	17.3
Six4	Kidney failure, chronic	0	17.1
Vti1a	Kidney failure, chronic	0	16.9
Ap2m1	Kidney failure, chronic	0	16.4
Ptch1	Kidney failure, chronic	0	12.45
Vsx2	Kidney failure, chronic	0	2.65

### TGF-β1 Induces Upregulation of miR-342-5p and Downregulation of FoxO3 and Ptch1 in TCMK-1 Cells

To determine whether miR-342-5p, FoxO3, and Ptch1 correlate with the progression of RF, we measured the expression levels of miR-342-5p, FoxO3, and Ptch1 in TCMK-1 cells treated with TFG-β1. The results showed that miR-342-5p expression levels were gradually increased, while those of FoxO3 and Ptch1 decreased in a time-dependent manner (Figures 5C–E). Of note, FoxO3 and Ptch1 expression levels were the most significantly reduced at the 4-h time point. As demonstrated by FISH, miR-342-5p was mainly distributed in the cytoplasm of TCMK-1 cells, as was FoxO3 and Ptch1. The merged images showed that miR-342-5p was partially co-localized with Ptch1 (Figure 5F), indicating a potential interaction between them. These data indicated that miRNA-342-5p, FoxO3, and Ptch1 may be directly involved in the development of RF.

### Ptch1 Expression Is Regulated by miR-342-5p and FoxO3 in TCMK-1 Cells Treated With TGF-β1

**FIGURE 6:**
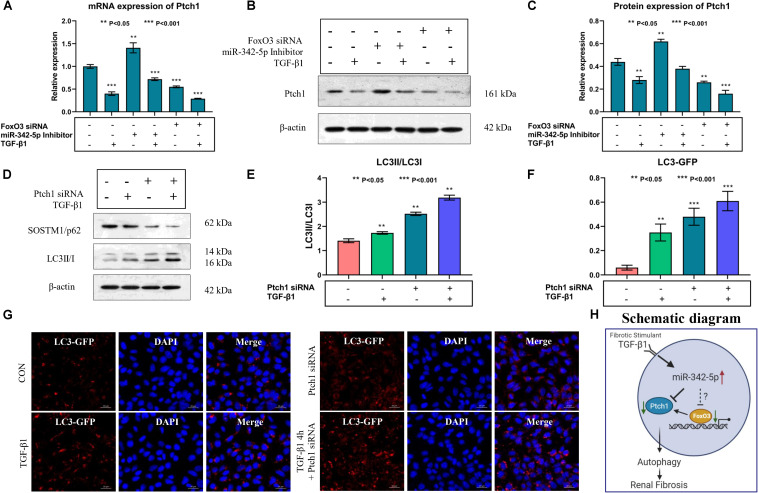
Downregulation of Ptch1 induced autophagy in TCMK-1 cells. **(A–C)** Real-time PCR and western blot showing that downregulation of miR-342-5p reverses TGF-β1-mediated inhibition of the expression of Ptch1 in TCMK-1 cells, while the downregulation of FoxO3 promotes TGF-β1-mediated inhibition of the expression of Ptch1 in TCMK-1 cells. **(D,E)** Western blot showing that knockdown of Ptch1 in TCMK-1 cells increased expression of SOSTM1/p62 and LC3II/LC3I. **(F,G)** Quantification of immunofluorescent LC3-GFP puncta showing that Ptch1 knockdown enhanced autophagy, as evidenced by an increase in the number of GFP-LC3 spots. **(H)** A simple schematic diagram showing the relationship between miR-342-5p, FoxO3, and Ptch1 in the progression of RF, and the role of autophagy in RF.

Real-time PCR and western blotting analyses showed that the expression levels of Ptch1 were regulated by miR-342-5p and FoxO3 in TCMK-1 cells treated with TGF-β1. Downregulation of miR-342-5p reversed the inhibitory effect of TGF-β1 treatment on Ptch1 expression in TCMK-1 cells, while the knockdown of FoxO3 gene exacerbated the TGF-β1-mediated inhibition of Ptch1 expression in TCMK-1 cells (Figures 6A–C). These data suggest that miR-342-5p negatively regulates Ptch1, which is transcriptionally regulated by FoxO3 during the progression of RF.

### Downregulation of Ptch1 Aggravated TGF-β1-Induced Autophagy in TCMK-1 Cells

Autophagy mediates the development of RF, and a previous study showed that Ptch1 could inhibit autophagy in Hedgehog (Hh)-dependent cancers (Chen et al., 2018). However, whether Ptch1 regulates autophagy and participates in the pathophysiological process of RF is not yet clear. Western blot analysis showed that the miR-342-5p inhibitor could reverse TGF-β1-induced downregulation of Ptch1 expression, while FoxO3 siRNA promoted the effect of TGF-β1 (Figures 6D,E). In addition, inhibition of Ptch1 promoted the TGF-β1-induced increase in LC3 II/I expression levels and also promote the TGF-β1-induced decrease in SQSTM1/p62 expression levels in TCMK-1 cells. In addition, we found that autophagy indicated by GP-LC3 spots increased after TGF-β1 stimulation. Downregulation of Ptch1 increased the number of GFP-LC3 spots (Figures 6F,G), indicating that inhibition of Ptch1 could exacerbate TGF-β1-induced autophagy in TCMK-1 cells. A schematic diagram showing the putative involvement of miR-342-5p, FoxO3, Ptch1, and autophagy in the progression of RF is shown in Figure 6H.

## Discussion

Renal fibrosis is the typical final pathological phenotype of all CKDs (Boor et al., 2010). Research on the mechanism of RF will contribute to a breakthrough in the treatment of CKD. In the past few years, even though the contribution of miRNAs to the pathogenesis of RF has been widely studied, the role of most miRNAs is not well understood. With the development of high-throughput sequencing analyses, an increasing number of studies have explored putative miRNAs that cause renal injury. Here, we evaluated an integrative network of miRNA and mRNA data to discover possible regulators of RF. In the present study, miR-342-5p was found to be a promising candidate miRNA affecting the progression of RF. KEGG pathway analysis of miR-342-5p showed that ECM receptor interaction is closely related to the pathophysiology of RF. As has been previously reported, the production of ECM proteins provided tissue scaffolds for normal repair events after renal injury (Lorena et al., 2002; Shen et al., 2016). Furthermore, researchers have found that miR-342-5p is upregulated more than sevenfold in paired samples of renal cell carcinoma tissue and adjacent non-tumorous renal parenchyma (Redova et al., 2013). On the contrary, Jiang et al., showed that miR-342-3p inhibited renal interstitial fibrosis in diabetic nephropathy as evidenced by a decrease in the levels of several biomarkers of RF [TGF-β1, FN, and collagen IV] (Jiang et al., 2020). As we know, two mature miRNAs, miR-342-3p and miR-342-5p, are excised from the same stem-loop pre-miRNA (Griffiths-Jones et al., 2006). “5p” and “3p” miRNAs are biologically different in functionality and stability, which is one of the reasons for the opposite results. Besides, the opposite results may be also due to different stimuli, different cell lines, and different animal models. Therefore, it is necessary to further study the role of miR-342-5p in RF.

During the past few years, many studies have shown that the expression levels of miRNAs and their downstream target genes are closely associated with the progression of RF (Li X.Y. et al., 2017; Yang et al., 2017; Chen, 2019; Liu et al., 2019). After applying various bioinformatics technologies as shown above, we found that Ptch1 is the most promising target mRNA of miR-342-5p in RF. Ptch1 is mainly expressed in epithelial cells around the renal tubules. Extracellular Hh ligands can bind to the Ptch1 receptor, allowing transmembrane protein Smoothened (SMO) to signal downstream and activate Glioma (Gli) TFs (Alexandre et al., 1996; Ruiz i Altaba, 1998). It has been shown that hypermethylation of Ptch1 is associated with the perpetuation of fibroblast activation and fibrosis in the liver (Yang et al., 2013). Similarly, inhibition of Ptch1 may have pro-fibrotic effects in kidney tissue by activating Hh signaling (Bai et al., 2014, 2016). Furthermore, Ptch1 gene mutations have been suggested to play an essential role in the pathogenesis of nephroblastoma (Isidor et al., 2012). Bioinformatics analysis predicted that miR-342-5p interacts with Ptch1 in the progression of RF. Our results indicated that TGF-β1 increases miR-342-5p and decreases FoxO3 and Ptch1 in TCMK-1 cells. The rescue experiment implied that Ptch1 expression was negatively regulated by miR-342-5p in TCMK-1 cells treated with TGF-β1. Thus, miR-342-5p is most likely to play a crucial role in the development of RF by regulating Ptch1.

Our bioinformatics results showed that FoxO3 is the TF of Ptch1. FoxO3 plays a critical role in various biological processes including development, proliferation, apoptosis, metabolism, and differentiation, by regulating expression of a wide spectrum of genes. It has been shown that FoxO3 may attenuate the idiopathic pulmonary fibrosis myofibroblast phenotype *in vitro* and bleomycin-induced lung fibrosis *in vivo* (Al-Tamari et al., 2018), prevent the progression of kidney diseases (Li et al., 2019; Lim et al., 2019) and promote kidney injury after UUO (Li L. et al., 2017). Hence, FoxO3 may play a role in promoting the fibrosis process by regulating Ptch1. FISH experiments indicated that miR-342-5p, FoxO3, and Ptch1 were mainly distributed in the cytoplasm of TCMK-1 cells. The merged images showed that miR-342-5p was partially colocalized with Ptch1. In line with the rescue experiment, miRNA-342-5p might activate fibrotic responses in the kidney by interacting with Ptch1, which is directly regulated by FoxO3. As reported previously, Ptch1 has therapeutic implications in Hh-dependent cancers by inhibiting autophagy (Chen et al., 2018). Autophagy can maintain cell homeostasis and energy under different physiological states (Klionsky, 2007). Quantification of immunofluorescent LC3-GFP puncta showed that inhibiting Ptch1 can enhance the effect of TGF-β1 on the promotion of autophagy. Therefore, the inhibition of Ptch1 could promote RF by activating TGF-β1 induced autophagy.

## Conclusion

Through bioinformatics analysis tools and *in vitro* experiments, we found that inhibition of Ptch1 regulated by miR-342-5p and FoxO3 could promote autophagy, which plays an essential role in the progression of RF. Further study is required to fully elucidate the role of miR-342-5p, Ptch1, and FoxO3 in RF. Nevertheless, our novel findings provide new insights into the pathogenesis and therapeutic targets of RF.

## Data Availability Statement

The data that support the findings of this study are downloaded from the GEO database (https://www.ncbi.nlm.nih.gov/geo/; GEO accession numbers: GSE118340, GSE42716, and GSE121190). All *in vitro* experiments data generated in this study are included in the article/Supplementary Material.

## Author Contributions

ST carried out the conception, derivation, and bioinformatics analysis of the study, and drafted the manuscript. YW generated the data from the GEO and analyzed the data preliminarily, and then helped to perform real-time PCR, western blotting. WL, GX, and JL conducted the cell culture and cell transfection. PL, YC, and FS collected and analyzed the numerical data. JZ guided the general research strategy and gave the critical revision of this manuscript. All authors read and approved the final manuscript.

## Conflict of Interest

The authors declare that the research was conducted in the absence of any commercial or financial relationships that could be construed as a potential conflict of interest.

## Footnotes

1 https://www.ncbi.nlm.nih.gov/geo/

2 http://www.ncbi.nlm.nih.gov/geo/geo2r

3 http://www.microrna.gr/miRPathv3/

4 https://david.ncifcrf.gov

5 http://mirwalk.umm.uni-heidelberg.de/

6 http://www.targetscan.org/mmu_72/

7 http://ctdbase.org

## Abbreviations

RF: renal fibrosis
ECM: extracellular matrix
miRNAs: MicroRNAs
UUO: unilateral ureteral obstruction
GEO: Gene Expression Omnibus
TF: transcription factor
DE: differentially expressed
GO: gene ontology
KEGG: Kyoto Encyclopedia of Gene and Genome
CTD: Comparative Toxicogenomics Database
CKDs: chronic kidney diseases
FISH: fluorescence *in situ* hybridization
Hh: hedgehog
SMO: smoothen.
